# Factors impacting the quality of peer relationships of youth with Tourette’s syndrome

**DOI:** 10.1186/s40359-015-0090-3

**Published:** 2015-09-30

**Authors:** Deirdre O’Hare, Valsamma Eapen, Edward Helmes, Kerry McBain, John Reece, Rachel Grove

**Affiliations:** Department of Psychology, College of Healthcare Sciences, James Cook University, Queensland, Australia; School of Psychiatry, University of New South Wales, Sydney, Australia; School of Psychological Sciences. Australian College of Applied Psychology, Melbourne, Australia

**Keywords:** Tourette, Peer relationships, Attachment, Personality

## Abstract

**Background:**

Tourette’s syndrome (TS) is a poorly understood neurodevelopmental disorder consistently associated with impaired peer relationships. This research aimed to investigate the relationship between TS and the ability of diagnosed youth to form secure attachment relationships with peers. A quantitative study examined differences between youth with TS and typically developing peers in social functioning, relationship problems and attachment security. Qualitative studies sought to identify factors that enhanced or impeded the ability to form secure peer relationships, including the impact of tic severity, comorbidity and personality traits. All research was conducted from the parental perspective.

**Methods:**

The research consisted of a controlled, survey-based qualitative and quantitative study (Study One) of parents of youth with TS (*n* = 86) and control group peers (*n* = 108), and a qualitative telephone interview-based study of TS group parents (Study Two, *n* = 22). Quantitative assessment of social functioning, peer problems and peer attachment security was conducted using the Paediatric Quality of Life inventory, the Strengths and Difficulties Questionnaire and the Attachment Questionnaire for Children. Qualitative data relating to personality was classified using the Five Factor Model.

**Results:**

Results revealed significantly higher rates of insecure peer attachment, problems in peer relationships, difficulty making friends, stigmatisation and lower levels of social functioning for the TS group. Significant between-group differences in number and type of factors impacting peer relationships were also determined with ‘personality’ emerging as the most prevalent factor. Whilst Extraversion and Agreeableness facilitated friendships for both groups, higher rates of Neuroticism were barriers to friendship for individuals with TS. The TS group also identified multiple ‘non-personality’ factors impacting peer relationships, including TS and comorbid symptom severity, the child’s psychological and behavioural adjustment to their disorder, coping strategies and the behaviour and attitudes of peers.

**Discussion:**

Our findings suggest that, whilst Extraversion and Agreeableness facilitated friendships for both groups, higher rates of Neuroticism were barriers to friendship for individuals with TS. Notwithstanding the fact that these findings are based on parental report and not the perceptions of youth themselves, this study may help clinicians to identify youth at increased risk of developing insecure peer relationships and guide the development of targeted supports.

**Conclusions:**

The findings from the study may help clinicians, parents and individuals with TS to better understand and cope with the difficulties experienced in interactions with peers.

**Electronic supplementary material:**

The online version of this article (doi:10.1186/s40359-015-0090-3) contains supplementary material, which is available to authorized users.

## Background

Tourette’s syndrome (TS) is a complex neurodevelopmental disorder characterised by the presence of multiple motor and vocal tics occurring for a period of at least one year [1]. The severity of TS varies widely between individuals and is complicated by high rates of comorbid diagnoses (90 %), the most common of which are Attention Deficit Hyperactivity Disorder (ADHD) and Obsessive Compulsive Disorder (OCD) [2]. Exhibiting a male gender bias of 3:1, the onset of symptoms typically occurs in childhood and peaks during the developmentally sensitive period of early adolescence [3].

Recent quality of life studies (QoL) on paediatric TS populations have revealed strong relationships between the disorder and decreased QoL, with the highest level of impairment evident in psychosocial functioning [4–11]. Further evidence suggests that TS has a particularly adverse impact on peer relationships. Prior research has found that youth with TS experience an increased incidence of bullying, teasing, peer victimisation and social rejection [11], have difficulty making and maintaining friends, have lower quality and numbers of close friends [12, 13] and are more likely to be negatively evaluated by peers [14, 15].

There is considerable individual variability in the level of difficulty youth with TS experience in their peer relationships and social functioning. However the factors contributing to these differences are not well understood. In previous studies, increased tic severity and the presence of comorbidity accounted for a significant proportion, but not all the variance in psychosocial outcomes [4–11]. Other factors with adverse effects on peer relationships include characteristics of TS such as impulsivity, aggressiveness, episodic rage and coprophenomena [16, 17] and the lower levels of social competence that some youth with TS exhibit [18]. Stigmatisation and social rejection also create limited opportunities for friendship and the development of social skills [6, 19], and diagnosed youth may limit their interaction with peers in response to fears associated with their own socially inappropriate symptoms or the negative behaviour of others [20].

The emergence of peer relationships as a key determinant of a wide range of outcomes for youth in recent TS studies highlights the importance of improving current understanding regarding the way in which TS impacts friendships. Having supportive and accepting friends has been associated with increased wellbeing, improved socio-emotional functioning and improved school performance [11, 21–24]. Conversely, negative peer behaviours and social isolation have been linked to higher rates of mood disorder, loneliness, poor self-esteem, self-consciousness and increased tic severity [11, 25]. The major goal of the current research was to develop a greater understanding of the peer relationships of youth with TS, and how TS itself may shape these relationships. As Attachment Theory has become the dominant model within which close relationships are examined [26], it was adopted for the purposes of the current exploratory study.

Ainsworth [27] and Bowlby [28] proposed that a classifiable style of attachment (secure or insecure) is developed during an infant’s interactions with their primary caregiver. This attachment style remains relatively stable across time and guides both expectations and behaviour in future relationships [27–30]. Secure attachment is contingent upon the primary caregiver’s positive representation of the child, their availability to provide a reliable source of safety and comfort in times of distress and a secure base for their child [27, 28]. As children develop, they gradually transfer these attachment functions from parents to peers, in a process that culminates in the development of romantic relationships and close friendships in adulthood [29, 31–33].

The literature reveals the critical role that the security of the child’s attachment relationships play in determining optimal development, childhood and future wellbeing [27–29], with secure attachment emerging as the strongest predictor of the child’s emotional and social competence. The relationship between attachment style and TS has not been explored in any prior published studies. Given the aforementioned psychosocial and peer relationship difficulties experienced by youth with TS, the current study hypothesised that youth with TS would be at increased risk of forming insecure relationships with peers.

In order to achieve the goals of the current research, two complementary studies that adopted a mixed method approach were conducted. Study One included a nation-wide survey-based study of parents of youth with TS (*n* = 86) and a group of parents of children without a diagnosis of TS (*n* = 108). The quantitative component of Study One examined differences in social functioning, problems in peer relationships and rates of insecure peer attachment between youth with TS, and age and gender matched peers. Given the novel and exploratory nature of this study, as well as difficulty identifying a multidimensional psychometric measure of peer attachment suitable for use across this age range, two qualitative studies were also conducted. Within Study One, qualitative data was gathered to identify factors that were perceived to enhance or impair the ability of youth to form secure peer relationships and to investigate differences in findings between the TS and control group. To augment these findings, a further interview-based qualitative study (Study Two) was conducted employing a subset of the TS group participants (*n* = 22) from Study One. Study Two aimed to collect qualitative data to identify the types of friendships experienced by youth with TS, as well as develop an understanding of motivation and other factors that shaped the security of peer relationships within this sample. Due to the ethical and practical implications of surveying children as young as seven within the design of the current study, information in both studies was provided by the primary caregiver, the majority of whom were the youths’ biological mothers.

As reported below, a key finding to emerge from the qualitative analyses related to the youths’ personality traits. Data relating to personality were classified according to the “Big-Five” Factor model (FFM) [34, 35], which consist of Extraversion, Agreeableness, Neuroticism, Conscientiousness and Openness (to experience). Previous research has found direct associations between Extraversion, Agreeableness and Openness and the ability to form and maintain friendships and wider social networks, and to develop social competence [36, 37], whilst Neuroticism has been found to have the opposite effect [37]. Personality traits may also indirectly affect peer relationships of youth with TS, with correlational studies demonstrating links between specific “Big Five” traits and a range of psychological and developmental disorders that adversely affect socio-emotional functioning. For example, autism has been correlated with low levels of Extraversion, Conscientiousness and Openness [38], while anxiety and depression have been consistently associated with Neuroticism [39]. The processes by which such links are established are, however, poorly understood. Furthermore, no research documenting the relationship between personality traits and TS has been published.

In addition, a broader literature exists regarding the impact of personality traits on resilience, coping abilities and strategies adopted by the individual in response to stressors, including those associated with chronic and developmental disorders, all of which may moderate or mediate the impact of TS on peer relationships. Extraversion, Agreeableness, Contentiousness and Openness have been associated with increased psychological resilience, in addition to improved problem focused coping and cognitive restructuring, whilst neuroticism has consistently predicted low resilience and maladaptive emotion-focused coping in youth [40]. In addition, personality traits influence the response of others, with agreeableness increasing an individual’s ability to enlist support and acceptance from others [41].

In summary, the current study sought to investigate the impact of TS on the peer relationships and the factors that enhanced or impeded their ability to experience secure peer relationships. Although primarily an exploratory study, several hypotheses were proposed. It was predicted that youth with TS would experience higher rates of insecure peer attachment, increased peer problems, and decreased social functioning in comparison with undiagnosed peers. It was further hypothesized that the qualitative analyses would reveal differences in factors identified by parents of youth with TS and controls as impacting their child’s ability to form secure relationships with peers, and that TS would be associated with unique barriers to secure peer relationships.

It is hoped that the findings from the current research have the potential to help clinicians, parents and young people with TS to understand the psychosocial difficulties of those diagnosed, particularly in the context of their peer relationships. It is also hoped that results have the potential to be employed to inform clinical intervention and encourage further research into this important aspect of TS youths’ psychosocial functioning.

## Methods

The research was conducted with the approval of the James Cook University Human Research Ethics Committee (Approval number H4380), in compliance with the Helsinki Agreement and within the guidelines for research ethics outlined in the National Statement on Ethics Conduct in Research Involving Humans (2007). Informed consent was obtained from all parents/guardians of participating minors in the study. Participation was confidential, all records were de-identified and stored in compliance with JCU guidelines, and no incentives to participate were offered.

### Participants

Two groups of volunteers participated in Study One (*N* = 194). The TS group (*n* = 86) consisted of a community based, national sample of parents with a child aged between 7 and 16 years formally diagnosed with TS. The control group (*n* = 108) comprised parents of age and gender matched peers with no reported medical or psychiatric diagnosis. A subset of TS group parents volunteered to participate in Study Two (*n* = 22).

### Procedure

**Fig. 1:**
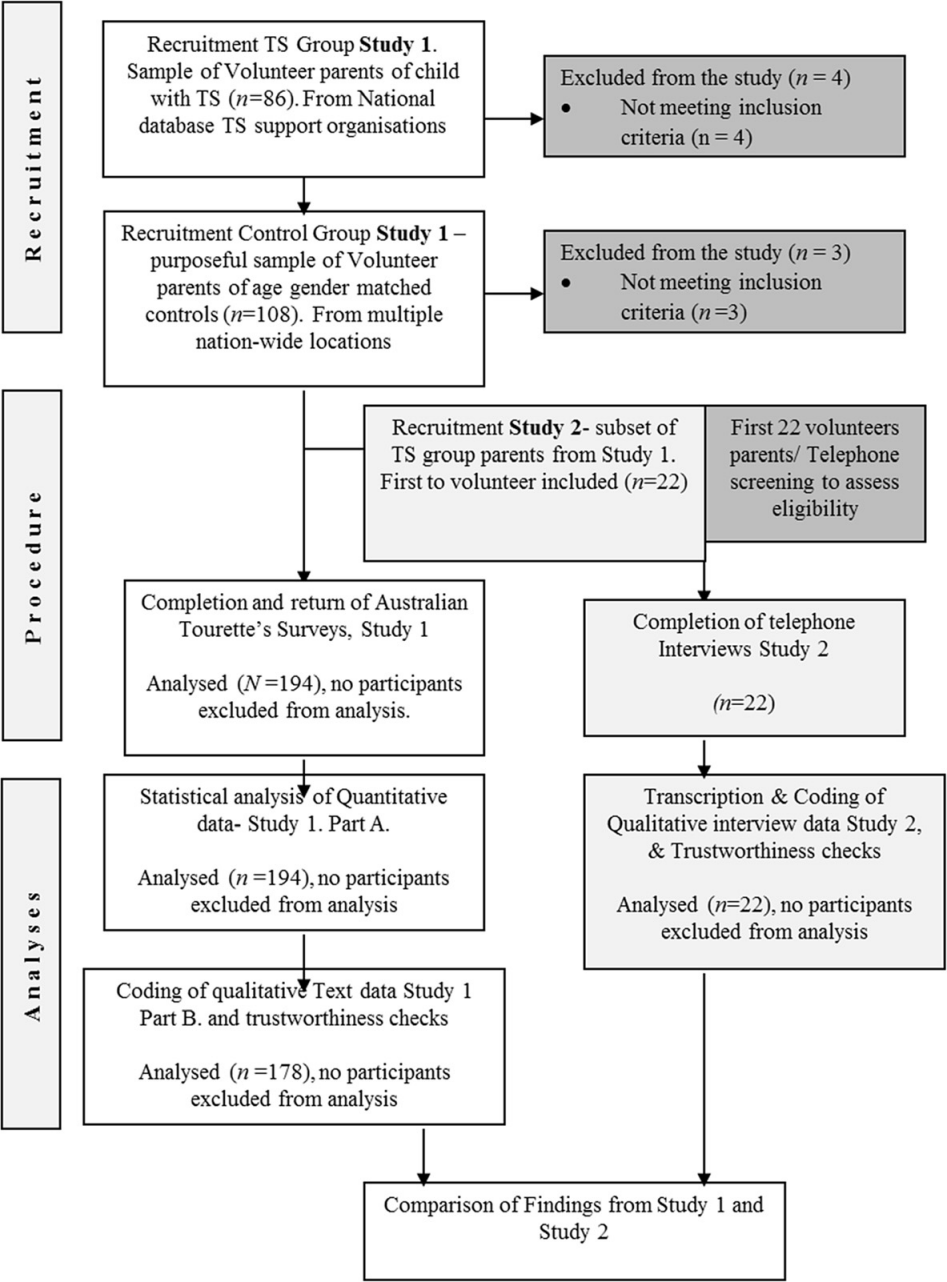
Flow Chart of the Research: Study 1 (Part A & Part B) & Study 2

TS group participants for Study One and Study Two were recruited with the assistance of the Tourette Syndrome Association Australia (TSAA) and the Tourette Syndrome Association Victoria (TSAV) following advertising and invitations to participate. Controls were recruited with the help of the TSAA and TSAV, research assistants in several nation-wide locations, and JCU University staff and students. Surveys were posted to the entire membership base of both societies, and mailed or distributed to controls. Accurate response rates could not be calculated due to the lack of information maintained on databases and the inability to track distribution of control group surveys. Figure 1 presents a flow chart of the present research.

### Measures

#### Study one

The Australian Tourette’s Survey (Additional file 1) was designed by the primary author for the purpose of a larger study of Australian youth with TS. In addition to demographic questions, the survey included questions that asked parents to identify their child’s formally diagnosed comorbid disorder and the experience of stigma. It also employed three widely used and well-validated psychometric measures (parent proxy versions) relevant to the current project to assess social functioning, peer relationship problems and security of peer attachment of youth in the study.

##### Social functioning

Social functioning was assessed via the social functioning subscale of the Pediatric Quality of Life Inventory (PedsQL) [42, 43]. This five-item subscale is a measure of quality of life related to social functioning. For example, parents are asked ‘How much of a problem has your child had (in the past month) getting along with other kids?’ Extensive reliability and validity data exist for the PedsQL [43–45]. Internal consistency for the social functioning subscale in the current study was excellent (Cronbach’s α = .90).

##### Peer relationships

Difficulties experienced in peer relationships was assessed via the peer problems subscale of the Strengths and Difficulties Questionnaire (SDQ) [46, 47]. This five-item measure forms part of a widely employed brief behavioural screening questionnaire assessing psychopathology, behavioural, and social problems. For example, parents are asked if the statement that their child ‘has at least one good friend’ (in the past 6 months) is not true, true or certainly true. The measure has robust psychometric properties [47–49]. Internal consistency in the current study for the peer problems subscale was excellent (Cronbach’s α = .87).

##### Peer attachment

The study adopted a broad operational definition of attachment that encompassed affectional (close or intimate) and non-affectional (friendships) components [50, 51]. This decision was guided by findings that have demonstrated the differing functions attachment figures perform at different developmental stages [52]. The availability of attachment figures to provide these functions at age appropriate times rather than the ‘closeness’ of the affectional bond is thought to be most relevant in the context of childhood and adolescent peer attachments [52, 53].

The single-item Attachment Questionnaire for Children (AQC) [54] was employed to assess peer attachment. The use of this age downward version of the widely employed single-item measure of self-reported adult attachment [55] was necessary due to the absence of multi-item measures of peer attachment suitable for use in middle childhood. Parents were asked to choose which of three narrative descriptions based on the three attachment styles constituting Ainsworth’s [56] typology (secure, insecure-ambivalent and insecure-avoidant) best described their child’s peer attachment. The reliability of the AQC and Hazan and Shaver’s measure has been demonstrated in multiple studies [54, 57, 58]. As the current research sought to determine the impact of secure versus insecure attachment, the two insecure attachment styles were combined to create a dichotomous variable evaluating secure or insecure attachment.

#### Qualitative studies

##### Study one

To gather data for the qualitative study, two questions were asked at the end of the demographics section of the survey. Parents were asked, “Does your child have any trouble making friends?” Participants were then asked to comment with a very brief written response to the question “What do you feel makes it easy or difficult for your child/teen to make friends?”

##### Study two

A series of open-ended questions were composed to form the basis of the semi-structured interviews that comprised Study Two. Questions were reviewed and refined by a senior academic supervisor before implementation. Example items include “How would you describe your child/teen’s friendships?” “How do you feel TS affects your child’s ability to make friends?” Questions were kept to a minimum to facilitate openness of responses from participants and to provide space for the participants’ voices and unique experiences [59].

All interviews were conducted via telephone by the principal researcher once consent had been obtained. Interviews varied in duration from one hour to 90 min. Participation was limited to one interview per parent, one parent per family, and one child or adolescent with TS. Each recorded interview was then transcribed in full for the purposes of data analysis.

### Coding of qualitative data

#### Study one

The principal researcher conducted a content analysis of the survey, employing a method described by Biddle and colleagues [60]*.* A deductive approach to coding was adopted, with the text read multiple times by the researcher to identify major themes, which were code-named as they emerged*.*

Data were further reduced by classifying each item related to the youth’s personality according to the “Big Five” (FFM) traits of extraversion, agreeableness, openness, conscientiousness and neuroticism [34, 35]. The validity of the findings regarding personality classification was examined by conducting third party trustworthiness checks (by a rater blind to the purposes of the study) on all of the coded personality data. This resulted in 100 % agreement.

#### Study two

The researcher employed both an inductive approach to detect emerging themes, as theoretically described by Strauss and Corbin [61], and a deductive approach to content analysis (guided by open-ended research questions grounded in the relevant literature) as described by Miles and Huberman [62]. The major analytic category of the current report was parental perception of the child’s peer attachments. Manual coding of verbatim transcripts was conducted with multiple line-by-line reviews to create a hierarchy from lower to higher order themes [60]. Manual coding with the aim of data reduction continued in an iterative process over multiple months until completion. Data dictionaries, which provided definitions and examples of all of the emerging codes, were composed for each study.

#### Inter-rater reliability

Two trained raters blind to the purposes of the study conducted inter-rater reliability checks. Using three randomly selected cases from each study, both raters checked all of the coded text data that emerged from Study One and all of the coded interview data from Study Two by referencing the respective data dictionaries. Percentage of agreement and Krippendorff’s alpha coefficient- KALPHA [63] were calculated using ReCAL [64]. 100 % agreement was determined for the overall sample, and all of the examined codes in Study One and Study Two achieved 100% agreement with KALPHA α =1. The high degree of agreement between raters may have been attributable to the clarity and simplicity of the variables in the study, which required minimal interpretation.

#### Statistical procedure

Data were analysed using SPSS Version 19 (IBM [65]). All continuous outcome and predictor variables were assessed for their suitability for parametric analyses by testing for normality and homogeneity of variance (Levene’s test). This was shown to be adequate for all variables. Outliers were examined and a per comparison critical significance level of α = .01 was applied for all comparisons. For all inferential test results, an *R*^2^-type effect size measure is reported (i.e., η_p_^2^ for ANOVA analyses).

In Study One, differences between the TS and control groups on the PedsQL and the SDQ subscales were evaluated with univariate ANOVAs. Chi-square analysis was used to test for significant relationships between group membership and responses to the AQC, demographic variables and the ability to form friendships.

The qualitative findings from both studies were cross-tabulated to generate frequency data. Z-tests of proportions were conducted to reveal between group differences in the qualitative findings from Study One, with a critical significance level of α = .01.

## Results

### Participant demographics

TS group parents (*n* = 86) and Controls (*n* = 108) participated in the quantitative components of Study One (*n* = 196). All TS group and 92 control group parents provided text for the qualitative analysis (TS, *n* = 86; Controls, *n* = 92; total, *n* = 178). The majority of participants in Study One were the biological mothers of the child or adolescent TS group = 91 %, Controls = 89 %), resided in metropolitan areas (TS group = 59 %, Controls = 62 %), were married (TS Group = 84 %, Controls = 82 %) and reported an average or above level of income (TS Group = 67 %, Controls = 70 %). Mean age of the children for the total sample was 11 (*SD* = 3 years), (TS Group, *M* =11.44, *SD* = 2.78; Controls, *M =*11.31, SD = 2.58). The majority of the youth included in the study were male (TS Group = 85 %, Controls = 73 %). Only one significant demographic difference was reported, with a slightly higher level of racial diversity reported within controls (*p* < .01).

Study Two participants included the child’s biological mother (*n* = 22, 100 %), were mostly married (*n* = 18, 82 %) and resided in metropolitan areas (*n* = 13, 59 %). The mean age of the children in Study Two was 12 (*SD* = 3 years). 90 % of the youth in Study Two were male (*n* = 20).

### Quantitative findings

#### Study one

The results of the main quantitative analyses for Study One (presented in Table 1 and Table 2) support the hypotheses that youth with TS experience higher rates of parent reported peer problems, impaired social functioning, insecure peer attachment and difficulty forming friendships than control group peers. Almost half (*n* = 37, 45 %) of the TS group believed their child was stigmatised by their TS and a high rate of comorbidity was reported for the TS group (*n* = 66, 77 %). The most commonly reported co-morbidities were OCD (44 %), other anxiety disorders (36 %), ADHD (33 %) and Learning Disorder (LD) (19 %).

**Table 1:** Descriptive Statistics and Analysis of Variance Results for TS and Control Group parents on Social Functioning and Peer Problems

	Groups				Anova			
	TS Group (*n* = 86)		Control Group (*n* = 108)					
Outcome Measure	*M*	*SD*	*M*	*SD*	*F*	*df*	*p*	η_p_^2^
Social Functioning (PedsQL)	60.76	24.76	87.73	14.92	84.74	1, 192	< .001	.31
Peer Problems (SDQ)	3.05	2.41	.87	1.37	62.91	1, 192	. < .001	.25

**Table 2:** Descriptive and Chi-Square Results for Differences between TS and Control Groups in Attachment Security and Ability to Form Friendships

Variable	Group		*χ* ^2^			
	TS Group (*n* = 86)	Control Group (*n* =108)	Total	*χ* ^2^	df	*p*
Attachment (AQC)						
Secure	49 (57 %)	102 (94 %)	151 (78 %)	36.5	1	<.001
Insecure	37 (43 %)	6 (6 %)	45 (23 %)			
Difficulty Forming Friendships				37.3	1	<.001
Yes	33 (38 %)	4 (4 %)	37 (19 %)			
No	53 (62 %)	106 (96 %)	159 (81 %)			

### Qualitative findings

#### Characteristics of the friendships of youth with TS

Coding and analysis of data in Study Two provided maternal descriptions of the friendships of the current sample of youth with TS (*n* = 22), with findings suggesting fewer than 20 % enjoyed a ‘typical’ social life. Mothers defined ‘typical’ as having at least one close friend, several peripheral friends and the ability to socialise with classmates and acquaintances. Mothers attributed reduced motivation for peer interaction to factors associated with TS symptoms including social anxiety (*n* = 3*,* 14 %), fear of bullying, teasing and rejection (*n =* 4, 18 %), difficulty maintaining friendships (*n =* 5*,* 23 %), inability to spend long periods of time with friends due to efforts to supress tics (*n =* 6*,* 27 %) and having a low level of interest in classmates (*n* = 2*,* 32 %). Almost a third of the youth in the sample had overtly expressed the desire for more friends to their mother (*n =* 7, 32 %). Motivation for friendship and romantic relationships was reported to increase for three of the four older adolescents.

#### Factors impacting peer attachment – parental perspectives

Findings from both studies revealed multiple factors impacting the peer relationships of youth. Parents in Study One identified twenty-two factors; The first being ‘Personality’ (FFM Traits) followed by twenty-one discrete ‘Non-Personality’ factors. Study Two revealed eighteen factors including ‘Personality’ that enhanced peer attachment and seventeen that negatively impacted peer attachment. There was a high degree of homogeneity across the findings from both studies in the factors identified by parents of youth with TS.

#### Between group differences in factors impacting peer relationships

As hypothesised, Study One revealed variability in the type, frequency and number of factors identified by TS and control parents affecting their child’s peer relationships. ‘Personality’ (FFM Traits) emerged as the most frequently identified factor for both TS and control group parents (see Table 3). The analyses of data from Study One also revealed the increased complexity of the factors identified by TS group parents in comparison with controls. No significant difference was found between the proportion of parents in the two groups who made reference to personality factors (TS = 74 %, control group = 88 %, *z* = 2.13, *p* < .05). However, a significantly larger proportion of parents in the control group (37 %) attributed the ability to form friendships exclusively to their child’s personality compared with the TS group (11.6 %) (*z* = 4.14, *p* < .001). TS group parents (88 %) identified a significantly increased number of ‘non-personality’ factors compared with controls (63 %) (*z* = 4.14, *p* < .01).

**Table 3:** Frequency and z-Test of Proportions between TS youth and Control Groups in Personality Traits with Positive and Negative Impact on Friendship

Personality Trait With Positive Impact	Group		Test of Difference in Proportions (two-tailed)	
	TS Group	Control Group	*z*	*p*
	*n* = 86	*n* = 92		
	*n* (%)	*n* (%)		
High Extraversion	48 (56)	84 (91)	5.77	< .001
Low Neuroticism	9 (11)	45 (49)	6.18	< .001
High Agreeableness	24 (28)	42 (46)	2.48	.01
High Openness	15 (17)	15 (16)	0.19	.85
High Conscientiousness	0	2 (2)	1.40	.16
Personality Trait With Negative Impact				
Extraversion	8 (19)	1 (1)	4.12	< .001
Introversion	10 (12)	12 (13)	0.28	.77
High Neuroticism	25 (29)	1 (1)	5.55	< .001
Agreeableness	0	1 (1)	1.01	.31
Low Agreeableness	12 (14)	2 (2)	2.89	.004
Low Openness	0	5 (7.7 %)		
Conscientiousness	0	4 (4)	2.02	.04
Low Conscientiousness	0	3 (3)	1.76	.08

As presented in Table 3, the major findings for ‘Personality’ were that Extraversion, low Neuroticism and Agreeableness had positive impacts for both groups, but were identified by a significantly higher percentage of control group parents. Extraversion, Neuroticism and low Agreeableness were associated with significant negative impacts for the TS group (see Table 3). The major findings for non-personality factors (Table 4) included the positive role of high Social Skills and Activities for controls in comparison with TS group youth, and the ability to cope with tics, a Positive School Environment and the Positive Behaviour of Others benefiting the friendships of TS group youth. The main negative ‘Non-Personality’ factors for TS versus control youth included Maladaptive Symptoms, the Negative Impact of Tics, low Social Skills, and the Negative Behaviour of Others (see Table 4).

**Table 4:** Frequency and z-Tests of Proportions Between TS Youth and Control Group in Non-Personality Factors with Positive or Negative Impact on Friendship

	Positive Impact				Negative Impact			
“Non – Personality” Factors	Group		Test of Difference in Proportions (two-tailed)		Group		Test of Difference in Proportions (two-tailed)	
	Control Group	TS Group	*z*	*p*	Control Group	TS Group	*z*	*p*
	(*n* = 92)	(*n* = 86)			(*n* = 92)	(*n* = 92)		
Social skills - High	14 (17)	-	4.30	< .001	-	-		
Social skills - Low	-	-	-	-	1 (1)	20 (23)	4.69	< .001
Social interest - Low	-	-	-	-	2 (2)	3 (4)	0.52	.6
Sports participation	18 (20)	9 (11)	1.71	.08	1 (1)	-	1.01	.3
Humour	7 (8)	2 (2)	1.65	.10	-	-	-	-
Activities & interests	11 (12)	2 (2)	2.57	.01	-	-	-	-
Parents	14 (15)	5 (6)	2.07	.04	-	-	-	-
School - Positive	-	7 (8)	2.73	.01	-	-	-	-
School - Negative	-	-			2 (2)	5 (6)	1.21	.2
Maladaptive symptoms	-	-			-	46 (54)	9.89	< .001
Tics - OK	-	17 (20)	4.58	< .001	-	-	-	-
Tics - Negative	-	-	-		-	20 (23)	5.07	< .001
Negative behaviours from others	-	-	-	-	1 (1)	23 (27)	5.20	< .001
Positive behaviours from others	-	9 (11)	3.24	.01	-	-	-	-
Understanding & acceptance	-	4 (5)	2.02	.04	-	-	-	-
Lack of understanding & acceptance	-	-	-		-	7 (8)	2.74	.006
Opportunity to socialize	5 (5)	1 (1)	1.59	.1	-	-	-	-
Child as “different”	-	-	-	-	-	8 (9)	2.95	.003
Age-passage of time - Positive	1 (1)	7 (8)	2.23	.02	-	-	-	-
Age-passage of time - Negative	-	-	-	-	2 (2)	6 (7)	1.52	.12
Preference for one or a small group friends	4 (4)	4 (5)	0.10	.9	-	4 (5)	2.02	.04
Trouble maintaining friendships	-	-	-	-	-	5 (6)	2.29	.02
Having long-term friends	1 (1)	3 (4)	1.06	.29	-	-	-	-
Preferring older or younger friends; not peers	1 (1)	2 (2)	0.61	.54	-	-	-	-
Context	2 (2)	2 (2)	0.04	.96	2 (2)	-	-	-
“Alike” kids	1 (1)	2 (2)	0.61	.54	-	-	-	-
Sibling	4 (4)	1 (1)	1.27	.20	-	-	-	-

Note. Raw scores indicate frequency of references made to the “Other” factor. Total positive or negative impact attributable to each factor by group membership expressed as raw score and percentage

## Discussion

To the best of our knowledge, this is the first study to explore the ability of TS diagnosed youth to form secure relationships with peers. The quantitative results from Study One, a large community-based survey of parents of youth with TS and age and gender matched peers, confirmed the hypothesis that youth with TS are at increased risk of forming insecure peer attachment relationships. Between group differences in the measure of peer attachment security reveal a threefold increase in insecure peer attachment for youth with TS, with the rate of insecurity for those diagnosed exceeding that expected in a normative population sample [27].

Additional quantitative results from Study One illustrate the adverse consequences of TS for the peer relationships of diagnosed youth. As hypothesized, peer relationships are likely to be negatively influenced by the highly significant elevation in impaired social functioning parents reported for youth with TS in comparison with controls. This finding has been reported in several recent studies of TS [6,8, 9, 66]. Consistent with previous research [11–13], parental reports confirmed that youth with TS experience a greatly increased number of problems (such as bullying and social rejection) within their peer relationships, and increased difficulty forming friendships in comparison with undiagnosed peers. Almost half of the parents in the TS group believed their child to be stigmatised by their diagnosis. This aligns with the rates reported in recent studies [6], and indicates barriers to positive peer relationships occurring at the societal level.

It is however important to note two significant limitations associated with the design of the current research when interpreting these findings. First, the study was limited to parental responses due to the ethical and practical constraints associated with surveying children as young as seven in remote mode (i.e., written survey and telephone interview). Second, in order to maximise participation from the difficult to access TS population, the study included a wide age range of youth, and therefore was unable to adequately control for the developmental stage of the youth under study.

### Characteristics of the friendships of youth with TS

Whilst determining quantitative differences between the type or number of friendships youth with TS and peers experienced was beyond the scope of the current research, evidence from Study Two suggests the restricted nature of friendship this sample of diagnosed youth appears to experience. Further, with the exception of an increased motivation for friendship and romantic relationships amongst older adolescents in the study, the findings did not reflect any change in the nature of friendship that might be expected at different developmental stages. Friendships appeared to be largely limited to a circle rarely extending beyond one to three individuals, with the majority experiencing impaired or restricted interaction with classmates and wider peer acquaintances. While some attachment theorists suggest that peer attachment is most relevant in the context of the child’s close ‘best’ friends [67], others stress the importance of the attachment functions played by less intimate peer relationships and more extensive social networks [52, 53]. The results of the current study indicate that these wider social networks may not be available to the majority of youth in this study.

Whilst these qualitative findings are limited by the subjectivity of maternal beliefs regarding ‘typical’ friendships, participant’s definitions were highly consistent. Future studies should build upon these findings by employing objective measures to assess friendship, as well as be extended to include the youth’s self-reported interpersonal experiences.

### Factors impacting the peer attachment relationships of youth with TS

The combined qualitative findings from Study One and Study Two revealed multiple factors that parents perceived as impeding or enhancing their child’s ability to form secure peer relationships. These fell into two broad categories, those related to the FFM personality traits [34] (Table 3) and those representing a broad range of ‘non-personality’ factors (Table 4). As hypothesised, findings indicated substantial variability in the factors identified by parents of youth with TS and control group parents, as well as the barriers to friendship specifically associated with diagnostic status.

#### Non-personality factors

Parents identified many ‘non-personality’ factors that they believed impacted the quality of their children’s peer relationships. These included the adverse effects of increased tic severity and the presence of comorbid disorders on the peer relationships of youth, although standardised assessment of tic severity and the quantitative impact of these variables were not goals of the current study. This is consistent with previous research [4–8, 11, 66, 68].

Current findings also revealed a highly complex role for tics and increased tic severity in impairment in peer relationships. Whilst this research found that simply having tics and increased tic severity are detrimental to peer relationships, distress and dysfunction in peer relationships was more closely related to the youth’s negative cognitive appraisal and their affective and behavioural responses to their tics. Specifically, the degree of self-consciousness experienced in regard to tics, rather than tic severity, appears to be most damaging to the youth’s peer relationships. This is an important finding as it may help to explain some of the individual variability in results of the impact of tic severity on social functioning evident in prior studies.

In addition, parents in Study Two linked increased self-consciousness and an inability to adjust to or accept a diagnosis with highly adverse behavioural and psychological consequences that further alienated individuals with TS from their peers. These included responses such as denial, rage, depression, social anxiety and social withdrawal. Supporting the acuity of these parental observations is the finding that a diagnosis of chronic disorder places youth at a significantly increased risk of adjustment disorder [69].

Parents also identified three more factors with negative impacts directly attributable to the ‘non-tic’ symptoms of their child’s TS and comorbid diagnoses. These included maladaptive symptoms of TS such as aggressiveness, impulsivity, a tendency to dominate peers, to behave bizarrely, incongruently, or to withdraw from or fail to participate socially. These factors have all been shown to have notable adverse effects on interpersonal relationships in previous TS studies [16, 17].

Parents also indicated that some of the behaviours associated with comorbid diagnoses, including inattention, impulsivity, anxiety, obsessiveness, defiance and antisocial behaviour had a significant impact on their child’s relationships. High rates of comorbid diagnoses were evident in both studies. Disorders such as OCD and ADHD have previously accounted for a disproportionate amount of social adversity for individuals with TS [4]. It is however important to note that disentangling behaviours attributable to TS from comorbid disorders, the child’s underlying personality traits, and behaviours that would generally be construed as misbehaviour, was reported as being very challenging for parents in the current study (Study Two). This is also a challenge in both clinical and research settings.

As identified in prior studies [6], negative peer behaviours such as bullying, teasing, social rejection and stigmatisation were reported as being a major impediment to secure peer relationships for many TS youth. The concerns of youth regarding peer responses towards them in this study also extended to a fear of being seen as “different”, “uncool”, “weird”, receiving unwanted peer attention and anxiety that they may frighten or irritate peers with their symptoms. For youth who experience socially embarrassing tics such as coprolalia, these self-perceptions are understandable. However, there is also some suggestion that some individuals with TS may “self-stigmatise” by internalising negative TS stereotypes and adverse social experiences, including being stigmatised by others [70].

Other less frequently identified factors to negatively affect peer relationships included poor social skills and competence, which parents often attributed to comorbid disorders and low social interest, that is a manifestation of the youth’s “eccentricity”, “shyness” or comorbidity. The ability to participate in sport was also important and appears to be of great cultural significance in the Australian context, particularly for males [71]. Non-participation for youth with TS appears to be associated with low interest, physical limitations associated with diagnosis, psychological barriers (e.g., social anxiety), the stress of competition and the youth’s cognitive rigidity. Parents also identified barriers to participation, such as social exclusion by peers, other parents, schools and social organisations.

Both studies also identified multiple ‘non-personality’ factors that enhanced the ability to form secure peer relationships. The most common factor was the youth’s ability to cope with their tics. This referred to a suite of factors including the youth’s successful psychological adjustment to the diagnosis of TS, as well as a lower level of self-consciousness in regard to their tic symptomology. Parents reported that the ability to camouflage, suppress or otherwise manage tics at important times (such as during class or whilst playing with other children), and being in a waning phase of the tic cycle both positively impacted the development of secure relationships with peers.

Other important positive factors included the youth’s ability to be open with others about their TS and comorbid disorders, to self-advocate and educate peers regarding their TS and their ability to defend themselves against the adverse behaviour of others (such as bullying teasing and social rejection). Some of these abilities form components of recent interventions designed to improve outcomes for children and adolescents with TS, particularly in the school setting [72, 73].

Finally, external factors including the acceptance, understanding and support of peers emerged as a key determinate of positive peer relationships, with parents in Study Two identifying this factor more frequently than any other. Some participants also acknowledged that peers needed occasional respite and support in order to preserve friendship with youth with TS. The importance of the attitudes of classmates to the relationships and wellbeing of youth with TS has been demonstrated during early trials of school-based interventions [73].

#### Personality factors

Personality was the most frequently identified factor impacting peer relationships to emerge from the current research, with over 80 % of Study One respondents attributing the quality of their child’s peer relationships to at least one personality dimension. Although there were no between-group differences in the frequency with which parents nominated personality, the control group were more likely to refer exclusively to FFM personality traits.

Of the FFM traits, Extraversion and Agreeableness, and to a limited degree Openness (to experience), appear to help youth with TS to overcome the significant barriers to friendship that they experience, as well as counterbalance the stigmatising, alienating and disturbing impact of their symptoms. As noted in the literature, Extraversion and Agreeableness have direct positive effects on peer relationships, with both linked to more positive peer representations. Extroverts are valued for their sociability, drive and energy, and this trait is the strongest predictor of friendships longitudinally [36, 37]. Those high in Agreeableness are appreciated for their caring, loving and empathic qualities, and Agreeableness predicts the highest rate of peer acceptance and reciprocity of friendship [36, 37].

Both Extraversion and Agreeableness may also indirectly enhance peer relationships by increasing resilience, adaptive coping and attracting social support. An extrovert’s assertiveness and social skills may help them to overcome the often reported negative behaviour of peers experienced by individuals with TS [6, 11, 15, 19], and enhance self advocacy skills. Agreeableness may help youth to recruit peer support and understanding [36, 37]. However, extroversion was also linked to problems with peers by some TS group parents. Furthermore, it has been suggested that the increased drive of extroverts for friendship may expose individuals high in this trait to increased psychological risk associated with rejection [74]. Openness to experience may extend the social networks of youth, which were found to be limited in this study, and the opportunities these provide for relationships facilitated by shared values and interests [36, 37, 75].

Consistent with the literature, the results of the current study indicated that Neuroticism was identified by parents as being commonly associated with the inability to form friendships in individuals with TS. It appears to share the closest theoretical link to insecure attachment, as both insecure attachment and Neuroticism are characterised by emotional dysregulation and negative affect [27, 28, 34, 35]. Previous research has also suggested that Neuroticism has the most adverse impact on the ability to maintain friendships [37], a problem that was identified for a significant minority of youth in Study Two. Emotional dysregulation, as well as a decreased ability to interpret social cues associated with Neuroticism, has also been shown to result in increased relationship conflict [76]. Finally, Neuroticism has been associated with decreased resilience and the impaired ability to cope with adversity in the context of chronic disorders [41], both of which may adversely affect social functioning and peer relationships.

### Limitations and future research

Several limitations have been noted for this study that may be addressed in future research. The generalisability of the results of the qualitative study may be limited by recruitment from TS support groups, the presence of unmeasured respondent characteristics, the demographic homogeneity of participants, and the dominant participation by biological mothers of male children and teens. Further, social desirability may have biased participants’ responses, while the researcher may have introduced bias at any or all levels of the qualitative analyses. Quantitative assessment of attachment was limited to a single-item measure due to apparent lack of availability of an appropriate multi-item measure for middle childhood. However, to some extent this was compensated for by the inclusion of the two qualitative studies.

A high priority is to compare the current findings with youth self-reports, which have been found to vary slightly from parent-reported outcomes in prior research on TS [6]. Future studies could also examine variability in the impact of TS on peer relationships at different developmental stages. The reliability and validity of the current qualitative findings would also benefit from replication in quantitative studies employing standardised measures of variables such as tic severity and psychopathology.

## Conclusions

The current study explored and confirmed the positive relationship between TS and the increased risk of developing insecure peer attachment relationships. Findings also provided detailed insights into multiple factors parents identified as either impeding or enhancing the development of effective peer relationships in youth with TS. These included the impact of TS and comorbid diagnoses, emotional, cognitive and behavioural response to diagnosis, the attitudes and behaviour of peers, as well as a number of personality traits.

The findings from the study may help clinicians, parents and those with TS to better understand and cope with the difficulties experienced in their interactions with peers. They may also help clinicians to identify those more at risk for developing poor peer relationships and guide the development of targeted supports, although it must be remembered that these findings are based on parental reports and not the perceptions of youth themselves.

Finally, the emergence of personality as an important variable suggests the value of including personality assessment in future research examining individual differences in youth with TS.

## Additional file

Additional file 1.
**Australian Tourette Survey.** (DOCX 356 kb)

## Abbreviations

ADHD: Attention deficit hyperactivity disorder
AQC: Attachment questionnaire for children
FFM: Five Factor Model
OCD: Obsessive compulsive disorder
PedsQL: Pediatric quality of life inventory
QoL: Quality of life
SDQ: Strengths and difficulties questionnaire
TS: Tourette’s syndrome
TSAA: Tourette Syndrome Association Australia
TSAV: Tourette Syndrome Association Victoria
