# Supplementary material for: Factors impacting the quality of peer relationships of youth with Tourette’s syndrome
Source: BMC Psychol. 2015 Sep 30;3:34. doi: 10.1186/s40359-015-0090-3 (PMC4589979; doi:10.1186/s40359-015-0090-3)
Supplement: Additional file 1. — Australian Tourette Survey. (DOCX 356 kb) [file 40359_2015_90_MOESM1_ESM.docx]

**PART A_________________________________________________________________________**

Participant ID: (Anonymous) Date:……………………

***Dear Participant,***

***Please complete all questions, by placing a Tic the appropriate box, or as a written answer as indicated.***

Example : 🗹 or please *write your answer* *on dotted line* ................

**Your Details**__________________________

**1**. Your Place of Residence: (Please Tic appropriate box)

Major city ☐ Regional town ☐

Rural or remote ☐

**2**. Your Relationship to the child / teen: (Please Tic appropriate box)

Biological mother ☐ Stepmother ☐ Adoptive Mother ☐ Other ☐

Biological father ☐ Stepfather ☐

Adoptive Father ☐ Other ☐

**3**. Your Marital Status (Please Tic appropriate box)

Never married ☐ Married /defacto ☐ Separated/divorced ☐

Widowed/widower ☐

**4**. Living situation: (Please Tic appropriate box)

Living with a husband/partner ☐

Single parent ☐ Other ☐

**5**. Number of children living with you (full or shared custody) (please write number here) ………………

**6**. What best describes your single parent or family income per year? (Please Tic appropriate box)

Up to $35,000 ☐ $35,000 – 75,000 ☐ Above $75,000 ☐

**7**. What race do you consider your child to be: (Please Tic appropriate box)

**Your Child’s Details___________________**

**8**.

Age of child = ……… years.

Gender: Male ☐ Female ☐

**9**. Has your child been diagnosed with Tourette’s Syndrome or Chronic Tic Disorder **(CTD)?**

(Please Tic appropriate box)

Yes ☐ No ☐

**10**. Does your child have any brothers or sisters?

(Please Tic appropriate box)

Yes ☐ No ☐

If yes, how many siblings? (Please write number here) ........................

**11**. Do any of your child’s brothers or sisters have Tourette’s Syndrome or CTD

(Please Tic appropriate box)

Yes ☐ No ☐

**12**. Has your child been diagnosed with any of the following disorders? (Please Tic appropriate box)

Attention/Hyperactivity disorder ☐Obsessive/compulsive behaviour/disorder ☐

Anxiety disorder ☐

Conduct disorder ☐

Impulse control ☐

Autism ☐

Learning disorder ☐

Depression ☐

Other (please write here)………………………

Caucasian ☐

Aboriginal /Torres Strait Islander ☐

Asian ☐ Other ☐

**13**. Do you think anyone else in your family may have or have had Tourette’s or CTD? (Please Tic appropriate box) Yes ☐ No ☐

**14**. Have any of your child’s brothers or sisters been diagnosed *by a professional* with any of the following disorders:

*(Please Tic appropriate box)*

Attention/hyperactivity disorder ☐

Obsessive/compulsive behaviour/disorder ☐

Anxiety disorder ☐

Conduct disorder ☐

Impulse control ☐

Autism ☐

Learning disorder ☐

Depression ☐

Other *(please write here)………………………………….*

**18.** Do you feel that your child is **stigmatized** as a result for having Tourette’s or CTD? *(Please Tic appropriate box)* Yes ☐ No ☐

**19**. Is your child receiving medication for his/her Tourette’s or CTD?

(Please Tic appropriate box) Yes ☐ No ☐

**20**. Is your child receiving any treatment or support from any of the following professionals? *(Please tic the appropriate box if Yes)*

Pediatrician ☐

Neurologist ☐

Psychologist (any type) ☐

Psychiatrist or child psychiatrist ☐

G.P. ☐

Counselor (school or other) ☐

Special education assistance ☐

Family therapist ☐

**15.** Do you find it difficult to access **medical** and/or **mental health services** for your son / daughter with Tourette’s or CTD?

*(Please Tic appropriate box)*

Yes ☐ No ☐

**16.** Do you feel that **medical** and **mental health professionals** know enough about Tourette’s or CTD and its treatment?

*(Please Tic appropriate box)*

Yes ☐ No ☐

**17.** Do you feel the **education system** is supportive of those with Tourette’s or CTD? *(Please Tic appropriate box)*

Yes ☐ No ☐

**21.** Does your child have any trouble making friends?

*(Please Tic appropriate box)*

Yes ☐ No ☐

**22.** What do you feel makes it easy or difficult for your child to make friends? *(Please write here) ……………………………………………………………………………………………………………………………………………………………………………………………………………………………………………………………………………………………………………………………………………………………………………………………………….*

*……………………………………………………….*

…………………………………………………………………………………………………………….

…………………………………………………………………………………………………………….

**Now please turn the page and continue with PART B**

**Part B:_____________________________________________________________**

**Please read the directions for each section of this survey carefully and answer *every* question as best you can. It is most important that you try to answer *every* question, even if you are not certain of your answer.**

**PLEASE BEGIN.**

**Directions**

Below is a list of things that **might** be a problem for **your child**. Please tell us **how much of a problem** each one has been for **your child** during the **past ONE month** by circling

**0** if it is **never** problem

**1** if it is **almost never** a problem

**2** if it is **sometimes** a problem

**3** if it is **often** a problem

**4** if it is **almost always** a problem.

There are no right or wrong answers.

If you are **not sure** of a response, **please give it your best estimate**.

It is **very important** that you please answer **all** items.

***Example:***

| 1. Walking more than one block | 0 | 1 | 2 | 3 | 4 |
| --- | --- | --- | --- | --- | --- |

**NEXT: Please answer ALL questions.**

In the **past ONE month**, how much of a **problem** has your child had with ……..

| Physical Functioning (problems with…) | ***Never*** | ***Almost never*** | ***Some-times*** | ***Often*** | ***Almost always*** |
| --- | --- | --- | --- | --- | --- |
| **1. Walking more than one block** | 0 | 1 | 2 | 3 | 4 |
| **2. Running** | 0 | 1 | 2 | 3 | 4 |
| **3. Participating in sports activity or exercise** | 0 | 1 | 2 | 3 | 4 |
| **4. Lifting something heavy** | 0 | 1 | 2 | 3 | 4 |
| **5. Doing chores around the house** | 0 | 1 | 2 | 3 | 4 |

| Emotional Functioning (problems with…) | ***Never*** | ***Almost never*** | ***Some-times*** | ***Often*** | ***Almost always*** |
| --- | --- | --- | --- | --- | --- |
| **1. Feeling afraid or scared** | 0 | 1 | 2 | 3 | 4 |
| **2. Feeling sad or blue** | 0 | 1 | 2 | 3 | 4 |
| **3. Feeling Angry** | 0 | 1 | 2 | 3 | 4 |
| **4. Worrying about what will happen to him or her** | 0 | 1 | 2 | 3 | 4 |

| Social Functioning (problems with…) | ***Never*** | ***Almost never*** | ***Some-times*** | ***Often*** | ***Almost always*** |
| --- | --- | --- | --- | --- | --- |
| **1. Getting along with other kids** | 0 | 1 | 2 | 3 | 4 |
| **2. Other kids not wanting to be his or her friend** | 0 | 1 | 2 | 3 | 4 |
| **3. Getting teased by other kids** | 0 | 1 | 2 | 3 | 4 |

| School Functioning (problems with…) | ***Never*** | ***Almost never*** | ***Some-times*** | ***Often*** | ***Almost always*** |
| --- | --- | --- | --- | --- | --- |
| **1. Paying attention in class** | 0 | 1 | 2 | 3 | 4 |
| **2. Forgetting things** | 0 | 1 | 2 | 3 | 4 |
| **3. Keeping up with schoolwork** | 0 | 1 | 2 | 3 | 4 |

**NEXT:**

For each item below, please mark the box for **Not True, Somewhat True** *OR* Certainly **True**. Please answer **ALL** questions as best you can even if you are not absolutely sure. Please give your answers *on the basis of your child’s behaviour over the past* ***six*** *months.*

| ITEMS  *Over the past 6 months, my child….* | Not  True | Somewhat  True | Certainly  True |
| --- | --- | --- | --- |
| Considerate of other people’s feelings | ☐ | ☐ | ☐ |
| Restless, overactive, can not stay still for long | ☐ | ☐ | ☐ |
| Often complains of headaches, stomach aches or sickness | ☐ | ☐ | ☐ |
| Shares readily with other children, for example toys, treats, pencils | ☐ | ☐ | ☐ |
| Often losses temper | ☐ | ☐ | ☐ |
| Rather solitary, prefers to play alone | ☐ | ☐ | ☐ |
| Generally well behaved, usually does what adults request | ☐ | ☐ | ☐ |
| Many worries or often seems worried | ☐ | ☐ | ☐ |
| Helpful if someone is hurt, upset or feeling ill | ☐ | ☐ | ☐ |
| Constantly fidgeting or squirming | ☐ | ☐ | ☐ |
| Has at last one good friend | ☐ | ☐ | ☐ |
| Often fights with other children or bullies them | ☐ | ☐ | ☐ |
| Often unhappy, depressed or tearful | ☐ | ☐ | ☐ |
| Generally liked by other children | ☐ | ☐ | ☐ |
| Easily distracted, concentration wanders | ☐ | ☐ | ☐ |
| Nervous or clingy in new situations, easily looses confidence | ☐ | ☐ | ☐ |
| Kind to younger children | ☐ | ☐ | ☐ |
| Often lies or cheats | ☐ | ☐ | ☐ |
| Picked on or bullied by other children | ☐ | ☐ | ☐ |
| Often volunteers to help others (parents, teachers, other children | ☐ | ☐ | ☐ |
| Thinks things out before acting | ☐ | ☐ | ☐ |
| Steals from home, school or elsewhere | ☐ | ☐ | ☐ |
| Gets along better with adults than with other children | ☐ | ☐ | ☐ |
| Many fears, easily scared | ☐ | ☐ | ☐ |
| Good attention span, sees chores or homework through to the end | ☐ | ☐ | ☐ |

Overall, do you think your child has difficulties in one or more of the following areas: Emotions, concentration, behaviour or being able to get on with other people?

*(Please tic appropriate box)*

No Yes- Minor difficulties Yes- Definite difficulties Yes- Severe difficulties

☐ ☐ ☐ ☐

*If you have answered* ***“Yes”****, please answer the* ***following questions*** *about these difficulties.*

- **How long have these difficulties been present?**

Less than a month 1 - 5 months 6 - 12 months Over a year

☐ ☐ ☐ ☐

- **Do the difficulties upset or distress your child?**

Not at all Only a Little Quite a lot A great deal

☐ ☐ ☐ ☐

- **Do the difficulties interfere with your child’s everyday life *in the following areas?***

Not at all Only a little Quite a lot A great deal

*HOME LIFE* ☐ ☐ ☐ ☐

*FRIENDSHIPS*  ☐ ☐ ☐ ☐

*CLASSROOM LEARNING* ☐ ☐ ☐ ☐

*LEISURE ACTIVITIES* ☐ ☐ ☐ ☐

- **Do the difficulties put a burden on you or the family as a whole?**

Not at all Only a Little Quite a lot A great deal

☐ ☐ ☐ ☐

**NEXT:**

Please choose **One** description from the **three options** below **(Tic One box only)** that **best describes your child,** even if it is not a totally accurate description.

***Description 1****.* ☐

*My son / daughter finds it easy to become close friends with other kids. My son/daughter trusts them and is comfortable depending on them. He / she does not worry about being abandoned or about another kid becoming too close friends with them.*

***Description 2.* ☐**

*My son / daughter is uncomfortable to be close friends with other kids. He /she finds it difficult to trust them completely, and it is difficult for him / her to depend on them. My son / daughter gets nervous when another kid wants to become close friends with him / her. Friends often come more close to my son / daughter than he / she wants them to.*

***(Please turn over for Description 3)***

***Description 3.* ☐**

*My son / daughter often finds that other children do not want to get as close as he / she would like them to be. My son / daughter is often worried that his / her friend doesn’t really like him / her, and that they may want to end their friendship. My son / daughter prefers to do everything together with his / her best friend. However, this desire sometimes scares other kids away.*

***NEXT:***

***Please Note: If your child has never had any tics, you are not required to complete the following section of this survey***

**“Tic Questionnaire”**

***Step 1.*** For **each** of the tics listed below, please mark “Yes” or “No” as to **WHETHER OR NOT your child has had the tic in the PAST MONTH**

***Step 2.*** For each tic you mark as **“Yes”,** please **circle** how **FREQUENTLY** the tic occurred over the past week according to the following:

**Constantly**, almost all the time during the day

**Hourly,** at least once per hour

**Daily,** at least several times per day

**Weekly**, just a few times or less

***Step 3.*** Under **INTENSITY**, rate how intense you believe the tic **FELT** to your child over the past week. For example, if it **was very mild**, like a weak twitch, that would be a **“1”.** A much **more forceful** tic that would be **very noticeable to others** and **may even be painful** would be rated as a **“3” or higher**. **Any** tic that would be **obviously noticeable to others** should be rated as at **least a “2”.**

*Example:*

| Eye blinking | Yes No | Constantly Daily  Hourly Weekly | 1 2 3 4 |
| --- | --- | --- | --- |
| Eye rolling/ darting | Yes No | Constantly Daily  Hourly Weekly | 1 2 3 4 |

***We will begin with your Child’s Motor Tics (that is, tics that involve some part of the body moving). Please answer every question.***

| **MOTOR TICS** | ***PRESENT***  ***Yes or No*** | ***FREQUENCY*** | ***INTENSITY***  ***0 – 4*** |
| --- | --- | --- | --- |
| Eye blinking | Yes No | Constantly Daily  Hourly Weekly | 1 2 3 4 |
| Eye rolling / darting | Yes No | Constantly Daily  Hourly Weekly | 1 2 3 4 |
| Head jerk | Yes No | Constantly Daily  Hourly Weekly | 1 2 3 4 |
| Facial Grimace | Yes No | Constantly Daily  Hourly Weekly | 1 2 3 4 |
| Mouth/ tongue movements | Yes No | Constantly Daily  Hourly Weekly | 1 2 3 4 |
| Shoulder Shrugs | Yes No | Constantly Daily  Hourly Weekly | 1 2 3 4 |
| Chest / Stomach Tightening | Yes No | Constantly Daily  Hourly Weekly | 1 2 3 4 |
| Pelvic tensing movements | Yes No | Constantly Daily  Hourly Weekly | 1 2 3 4 |
| Leg/ feet movements | Yes No | Constantly Daily  Hourly Weekly | 1 2 3 4 |
| Arm/hand movements | Yes No | Constantly Daily  Hourly Weekly | 1 2 3 4 |
| Copying others gestures | Yes No | Constantly Daily  Hourly Weekly | 1 2 3 4 |
| Obscene gestures | Yes No | Constantly Daily  Hourly Weekly | 1 2 3 4 |
| Other motor tics | Yes No | Constantly Daily  Hourly Weekly | 1 2 3 4 |
| Complex motor combinations (multiple tics at once) | Yes No | Constantly Daily  Hourly Weekly | 1 2 3 4 |

***Now let’s look at your child’s VOCAL TICS (any noises words or sounds made)***

**Please answer every question.**

| **VOCAL TICS** | ***PRESENT***  ***Yes or No*** | ***FREQUENCY*** | ***INTENSITY***  ***0-4*** |
| --- | --- | --- | --- |
| Grunting | Yes No | Constantly Daily  Hourly Weekly | 1 2 3 4 |
| Sniffing | Yes No | Constantly Daily  Hourly Weekly | 1 2 3 4 |
| Snorting | Yes No | Constantly Daily  Hourly Weekly | 1 2 3 4 |
| Coughing | Yes No | Constantly Daily  Hourly Weekly | 1 2 3 4 |
| Animal noises | Yes No | Constantly Daily  Hourly Weekly | 1 2 3 4 |
| Syllables | Yes No | Constantly Daily  Hourly Weekly | 1 2 3 4 |
| Words | Yes No | Constantly Daily  Hourly Weekly | 1 2 3 4 |
| Phrases (a few words together) | Yes No | Constantly Daily  Hourly Weekly | 1 2 3 4 |
| Repeating the words or sounds of others | Yes No | Constantly Daily  Hourly Weekly | 1 2 3 4 |
| Obscene or offensive words | Yes No | Constantly Daily  Hourly Weekly | 1 2 3 4 |
| Blocking/stuttering | Yes No | Constantly Daily  Hourly Weekly | 1 2 3 4 |
| Other | Yes No | Constantly Daily  Hourly Weekly | 1 2 3 4 |
| Other vocal tics | Yes No | Constantly Daily  Hourly Weekly | 1 2 3 4 |
| Complex vocal combinations (multiple sounds /words together) | Yes No | Constantly Daily  Hourly Weekly | 1 2 3 4 |

***The End***

- **Thank you so much for your participation**
- **Please check that EVERY question, on EVERY page has been answered, as best you can**
- **Please place the *completed questionnaire and your consent form* in the addressed envelop provided and mail it as soon as possible, or return your completed forms directly to the researcher. NO POSTAGE REQUIRED**

### INFORMATION SHEET

**“The Lives of Australian Children and Teens with Tourette’s Syndrome: A parent’s perspective”**

| You are invited to take part in a research project that aims to help psychologists to learn more about the lives of Australian children and teens with Tourette’s Syndrome (TS) or chronic tic disorder, conditions that have been understudied, particularly in Australia, as well as children who have neither of these experiences. As parents and primary caregivers, your knowledge and understanding of your child is of great value and importance. Parents and caregivers are being asked to share their understanding about their children to enable professionals to develop appropriate and improved services.  This study will be an important step towards building local knowledge about the Australian TS community and its needs. Deirdre O’Hare is conducting this research in completion of her Doctor of Clinical Psychology, at James Cook University, Queensland. | |
| --- | --- |
| If you agree to be involved in this study, you will be invited to complete a questionnaire that will ask for your observations of your child or teenager’s behaviours, emotions and strengths. This questionnaire should take approximately 20 - 30 minutes to complete. Once completed, you may return your questionnaire to the researcher in the stamped, addressed envelope provided. You will also be asked to indicate whether you are willing to participate in a follow-up interview scheduled at your convenience to discuss your views on these issues in more detail. | |
| Taking part in this study is completely voluntary and you can stop taking part in the study at any time without explanation or prejudice. You may also withdraw any unprocessed data from the study. | |
| Although no distress is anticipated from participating in this research, occasionally, people find certain items a little upsetting, If for any reason you become concerned whilst you are completing the questionnaire, or have any questions in regard to the questionnaire or the study, please advise Deirdre and you will be referred to someone who can help you. Deirdre’s phone and email details are provided below. In addition, 24 hour counselling assistance is available via LIFELINE by calling 13 11 14. | |
| If you know of others that might be interested in this study, please pass on this information sheet to them so they may contact Deirdre to volunteer for the study. The more parents that participate, the more we can learn. | |
| Your responses and contact details will be strictly confidential and your participation anonymous. The data from the study may be used in research publications. However, at no stage over the course of the research, or in subsequent publications, will either you or your son / daughter be identified. | |
| If you have any questions about the study, please contact **Deirdre O’Hare** or her supervisors,  **Dr Kerry Anne McBain or Professor Edward Helmes.**  **Thank you so very much for your time,**  Deirdre  **Deirdre O’Hare**  **Doctor of Clinical Psychology Candidate (James Cook University)** | |
|  | |
| **Principal Investigator:**  **Deirdre O’Hare**  **School of Humanities and Soc. Science**  **James Cook University**  **Phone: +61 (07) 4781 4706**  **Mobile: 0416 823 749**  **Email: deirdre.ohare@my.jcu.edu.au** | **Supervisor:**  **Dr Kerry Anne McBain, Prof Edward Helmes**  **Dr Beryl Buckby.**  **School of Humanities and Soc. Science**  **James Cook University**  **Phone: +61 (07) 4042 1207**  **Email: Kerry.mcbain@jcu.edu.au;Beryl.buckby@jcu.edu.au**  **Edward.helmes@jcu.edu.au;** |

### INFORMED CONSENT FORM

| PRINCIPAL INVESTIGATOR | **Deirdre O’Hare** | | | | |
| --- | --- | --- | --- | --- | --- |
| PROJECT TITLE: | **“ The lives of Australian Children and Teens with Tourette’s Syndrome or Chronic Tic Disorder: From their Parent’s Perspective”** | | | | |
| SCHOOL | **Humanities and Social Science** | | | | |
|  | | | | | |
| I understand the aim of this research study is learn more about the lives of Australian children and teens with Tourette’s Syndrome or Chronic Tic Disorder and their peers who do not have such experiences. As a parent of such a child / teen, I am being asked for my observations of my son or daughter’s behaviours and emotions. I consent to participate in this project, the details of which have been explained to me, and I have been provided with a written information sheet to keep. | | | | | |
| I understand that my participation will involve an interview and questionnaire and I agree that the researcher may use the results as described in the information sheet. | | | | | |
| I acknowledge that: | | | | | |
| - any risks and possible effects of participating in the ***interview and*** ***questionnaire*** have been explained to my satisfaction. It is not anticipated that my participation in this study will cause me any distress, however if I do experience any distress arising from completing this questionnaire, I may contact the researcher who will refer me to someone who can help | | | | | |
| - taking part in this study is voluntary and I am aware that I can stop taking part in it at any time without explanation or prejudice and to withdraw any unprocessed data I have provided; | | | | | |
| - that any information I give will be kept strictly confidential and that no names will be used to identify me with this study without my approval; | | | | | |
|  | | | | | |
| *(Please tick to indicate consent)* | | | | | |
| **I consent to be interviewed** | |  | **Yes** |  | **No** |
| **I consent for the interview to be audio taped** | |  | **Yes** |  | **No** |
| **I consent to complete a questionnaire** | |  | **Yes** |  | **No** |

| **Name:** *(printed)*  **________________________________________________________________________________**  **Signature: Date:** |
| --- |
